# Inebriation

**Published:** 1872-05

**Authors:** 


### INEBRIATION.

THERE is much food for parental thought in the last Report of the New York State Inebriate Asylum at Binghamton, N. Y., presented to the Legislature, January 1st, 1872, by D. J. Dodge, M. D., Superintendent and Physician. There is no family in the land who has not or may not have a personal interest in this admirably managed institution. A letter addressed to Carrol Hyde, Secretary, Binghamton, N. Y., will secure a report, which is full of interest. The object of this institution is to break up the habit of drinking liquor. Some of the statistics are suggestive. The number treated during 1871 was 315. Thirty per cent. of the inmates paid twenty dollars a week; twenty-five per cent. fifteen dollars; twenty per cent. could not pay anything; showing that more than one half of the inebriates are from the wealthier classes.

One fifth were discharged unimproved, meaning hopeless, utterly hopeless of reform; a terrible lesson to those who ever taste or touch the "flowing bowl."

One fifth had a collegiate education; two fifths had an academical education; all had been to school, showing that education of the intellect merely was no guarantee against a drunken life.

Two thirds were of a lively, cheerful disposition; of the temperaments, nervous, sanguine, bilious, each had a third. Two thirds were whiskey-drinkers; a fourth wine and brandy; fifteen from opium.

One third had intemperate ancestors. Two thirds drank habitually before meals. One fourth from trouble or pecuniary embarrassment.

One half were constant drinkers; one third had sprees now and then. One half were married; one third bachelors. The oldest, sixty-three; the youngest, nineteen.

One third were from the city. Nearly one half were merchants and merchants' clerks. One in twelve had no occupation. The chaplain reports that the inebriate is physically, mentally, and morally deteriorated; that he loses self-control, and that if left to himself to reform, he is, nine cases in ten, lost! because in the popular estimation there is little hope of his reformation; consequently all business confidence is withdrawn from him; he sees it; next he begins to be regarded with pity and contempt, and becomes conscious of his position; he loses all self-respect, becomes an outcast, and generally falls away into the darkness of a dishonored grave.

There were two clergymen, six physicians, and twenty-five lawyers. We visited Binghamton in August, 1871, purposely to become acquainted with the workings of the institution; but unfortunately there was no official in the house higher than the man who was sweeping the floor; but he did it well, and took a polite pleasure in showing and telling all he could; his views of inebriate management were thoughtful and philosophical. Little did he know the inexpressible sorrow a single sentence of his shot across our heart, when he said in answer to a question propounded in reference to an only son of one of our oldest, and best, and most cultivated and happy households, "Beyond remedy." Young, high-born, and heir to large estates, "beyond remedy." The institution is "beautiful for situation," commanding one of the loveliest views in the land. The rooms are large and airy. There are various pastimes, games, libraries, reading-rooms, music, regular public worship under the judicious conduct of Rev. Samuel W. Bush, Chaplain, whose report is worthy of circulation in every household.

The proceedings of the American Association for the Cure of Inebriates held its second meeting in New York city, in November, 1871. It gave an instructive Report of 115 pages, 8vo, naming several places opened for the reception of inebriates: the Washington Home, Boston, Mass., established in 1857, William C. Lawrence, Superintendent. Inebriates' Home for Kings County, Shore Road, Long Island, N. Y.; address Lewis D. Mason, M. D., Physician, Fort Hamilton P. O., New York. Washingtonian Home; address T. M. Vancourt, Superintendent, Chicago, Ill., 572 West Madison Street. Pennsylvania Sanitarium, under the efficient presidency of the distinguished and able physician and philanthropist, Joseph Parrish, M. D., Media P. O., Philadelphia, Pa. Greenwood Institute, Albert Day, M. D., Superintendent, whom address at Boston, Mass., No. 11 Tremont Temple. The institution is eight miles from Boston, on the Boston and Maine Railroad. Harlem Asylum for Inebriates, on Gilmore Street, near Franklin; address Dr. Casy B. Gambee, Medical Superintendent, Baltimore, Md.

There is a strong temptation to fill half a dozen pages with most important statements in reference to drunkenness taken from the Report of the American Association, of deep interest to parents, to physicians, clergymen, and the teachers and professors in all our schools, colleges, and universities, containing as it does essays on Inebriate Asylums in Great Britain. "The Social Tide of Temperance Reform," by a late inmate at Binghamton, New York. "Inebriety by Inheritance," by W. C. May, M. D. "Intemperance: the Disease and its Remedy," by Otis Clapp, Esq. "Pathology of Inebriety," by George Burr, M. D. "Classification and Treatment of Inebriates," by Joseph Parrish, M. D. "What Science and Inebriate Asylums have Taught us," by Willard Parker, M. D. "Experience at the Washingtonian Home at Boston," by the Superintendent, William C. Lawrence. "Hydrate of Chloral in Delirium Tremens," by Dr. J. A. Falette, seven cases. "The Superiority of State over Private or Smaller Institutions," by D. J. Dodge, M. D. "Brief Historical Sketch of the Chicago Washingtonian Home," by T. M. Vancourt, Superintendent.

These essays give an amount of inebriate literature, of standard character, which will add largely to the value of our public libraries, because it imparts the first information derived from the experience and observation of practical and educated men, in the conduct of the first Inebriate Homes, to be made use of for improvement in plan and management of all subsequently erected.

A very sad report comes from the Inebriates' Home at Fort Hamilton, New York, that nearly one half were women! Of seventy-two married, forty-five were separated from their partners because of intemperance. Two thirds of the whole number of inmates were separated from their partners, because of their intemperance. Four fifths were whiskey drinkers; one third hereditary drinkers. Beer-drinking Germans had not a single representative, nor had a single case of delirium tremens ever occurred in the institution as a result of drinking beer or other fermented liquors.

The reformed inebriate from Binghamton rejoices in the fact that instead of placing the bottle on the table to every visitor, which was almost universal less than fifty years ago, "It is no longer deemed obligatory to provide strong drink at christenings and funerals; that it is possible in New York city to have a wedding reception without wine and brandy; that some noble ladies have the courage on New Year's Day to place nothing stronger than tea and coffee before their callers." He closes by saying, "And last of all, and best of all, there exists an organized army of temperance men, who are determined to prohibit by law the sale of strong drink." This last sentence shows the sentiment of an educated man, who has been through the fire, that the most efficient engine for the prevention of intemperance is that which makes it most impossible to obtain anything to get drunk on. Prevention costs less than punishment and remedy. The philosophy, the theory, the practice of this whole subject of the banishment of intemperance as a national and social vice, is contained in half a dozen words of Holy Writ,

TOUCH NOT, TASTE NOT, HANDLE NOT.

